# Danggui buxue tang inhibited mesangial cell proliferation and extracellular matrix accumulation through GAS5/NF-κB pathway

**DOI:** 10.1042/BSR20181740

**Published:** 2019-10-18

**Authors:** Rui Zhang, Xiao Han, Tao Huang, Xiuge Wang

**Affiliations:** 1 Department of Endocrinology, The Affiliated Hospital of Changchun University of Chinese Medicine, Changchun 130021, Jilin Province, People’s Republic of China; 2 Department of Emergency Physicians, The Affiliated Hospital of Changchun University of Chinese Medicine, Changchun 130021, Jilin Province, People’s Republic of China

**Keywords:** diabetic nephropathy, GAS5, huangtao16125@163.com, mesangial cell, NF-κB

## Abstract

Diabetic nephropathy (DN) is the common complications of diabetes mellitus, but the efficacy of available treatments for the prevention of DN is still unsatisfactory. In the present study, we aimed to explore the effect of Danggui buxue tang (DGT) on the proliferation of high glucose (HG)-induced mesangial cells and accumulation of extracellular matrix in mesangial cells. We found DGT up-regulated the expression of growth arrest specific transcript 5 (GAS5) and IκB kinase (IKK) dose-dependently in mouse mesangial cells (SV40 MES-13). We found DGT regulated the expression IKK and the activity of nuclear transcription factor-κB (NF-κB) via GAS5, and proved that long non-coding RNA (lncRNA) GAS5 was positively related with IKK. And we proved GAS5 regulated the expression of IKK and the activity of NF-κB. In addition, DGT inhibited the viability of MES-13 cells and extracellular matrix-related proteins (laminin (LN), fibronectin (FN) and collagen IV (Col IV)) via GAS5. Moreover, we proved GAS5 regulated the viability of SV40 MES-13 cells and extracellular matrix-related proteins through NF-κB pathway. DGT inhibited the proliferation of mesangial cells and accumulation of extracellular matrix via GAS5/NF-κB, therefore, DGT could be an effective treatment for the prevention of DN.

## Introduction

Diabetic nephropathy (DN) is one of the most important and common complications of diabetes mellitus, which is characterized by persistent increment of urinary albumin or protein. It can result in the loss of renal function and lead to the failure of renal function [1,2]. The main pathological features of DN are renal interstitial fibrosis, nephropyelitis, renal papillary necrosis and renal arteriosclerosis. High glucose (HG, 20–30 mM) stimulates renal tubular epithelial cells and mesangial cells to produce massive reactive oxygen species (ROS), and induces the damage of renal tubulars and the change of extracellular matrix [3]. Currently, the main therapeutic strategies are tight control of blood glucose and blood pressure, low-protein diet, lipid-lowering and drug therapies [4,5]. However, the efficacy of available treatments for the prevention of DN is still unsatisfactory.

Danggui buxue tang (DGT) is a classic prescription containing two main ingredients, *Astragali Radix* (AR, active compound: astragalus polysaccharide, astragalus saponin) and *Angelica sinensis Radix* (ASR, active compound: ferulic acid, angelica polysaccharide, chlorogenic acid), which is originally formulated in Dong-Han in AD1247 of China. For thousands of years, it was commonly used for invigorating qi (qi is believed to be a vital force forming part of any living entity) and enriching blood [6]. It has been reported that DGT alleviates the progression of DN induced by streptozotocin (STZ) [7,8]. Researchers have found that DGT could attenuate extracellular matrix components, such as fibronectin (FN) or type IV collagen [9]. In glomerular mesangial cells, researchers have proved that DGT inhibited mesangial cell proliferation and the expressions of laminin (LN), FN and collagen IV (Col IV). However, the mechanism of DGT in the treatment of DN is still not clear.

Nuclear transcription factor-κB (NF-κB) pathway is a widely expressed transcription factor that involved in cell proliferation, invasion, metastasis, inflammation and angiogenesis [10]. Studies have shown that NF-κB plays critical role in DN. Lu et al. [11] reveled that polysaccharides relieved STZ-induced DN via regulating NF-κB pathway. Yang et al. [12] proved that HG induced the proliferation of mesangial cells in rat through the activation of NF-κB pathway. Moreover, Liu et al. [13] reported that DGT could reduce inflammatory damage in DN via NF-κB pathway. Hence, we speculated that DGT might inhibit the proliferation of mesangial cells via NF-κB pathway.

Long non-coding RNAs (lncRNAs) are more than 200 nt in length and emerged as important regulators in the biological processes [14]. LncRNA growth arrest specific transcript 5 (GAS5) is firstly discovered in T-cell lines and non-transformed lymphocytes in 2008. It is a critical tumor suppressor lncRNA in many cancers, such as breast cancer, colorectal cancer, non-small-cell lung cancer and hepatocellular carcinoma [15–17]. Recently, it has been reported that lncRNA GAS5 could inhibit the activity of NF-κB [18]. At the same time, lncRNA GAS5 was expressed in renal cell carcinoma cell line [16]. Therefore, we speculated that DGT might inhibit the proliferation of mesangial cells and accumulation of extracellular matrix by regulating NF-κB via GAS5.

## Materials and methods

### DGT preparation

DGT was consisted of ASR and AR at a ratio of 1:5 [19]. Slices of the herbs were purchased from Pharmacy in Changchun and the herbs were harvested in Shanxi province. The mixed herbs were extracted twice. First, ASR and AR were boiled together in water for 1 h (1:6; v/w). Then, the residue from first extraction was boiled in water for 1.5 h (1:8; v/w). Finally, the solutions were combined and filtered to remove insoluble debris. The filtered concentration of solution was 2.0 kg/l and stored at 4°C for the following experiments.

### Preparation of DGT-containing serum

Thirty male C57BL/6 mice (aged 7 weeks) were purchased from the animal center of Changchun university of Chinese medicine, and kept in a 12 h light/12 h dark environment with free access to water and food for 2 weeks. All animal experiments were approved by Ethic Committee of The Affiliated Hospital of Changchun University of Chinese Medicine. The mice were randomly divided into control (*n*=10) and DGT-containing serum (DGTXT) group (*n*=20). The DGTXT group was intragastrically administered DGT (20 g/kg.day) for 5 days, and the control was given distilled water of the same dose. All mice were anesthetized with pentobarbital (100 mg/kg, i.p., catalog number: P-010, Sigma, U.S.A.) [20,21] after 150 min since last intragastric administration, and blood samples were obtained from abdominal aorta. Loss of righting reflex, loss of palpebral reflex and absent pedal withdrawal response to pain were considered fully anesthesized. Serum specimens were centrifuged from blood samples, and then devitalized by water bath at 56°C for 30 min, filter sterilization with 0.22-μm filter, and stored at −80°C. Mice serum that did not contain DGT was considered as control.

### Cell culture and transfection

Mouse renal mesangial cells (SV40 MES-13) was purchased from Shanghai Cell Bank of Chinese Academy of Science, and cultured in 5.6 mM glucose Dulbecco’s modified Eagle’s medium (DMEM; catalog number: 21885108, Gibco, U.S.A.) supplemented with 10% fetal bovine serum (FBS; catalog number: 16140071, Gibco, U.S.A.) in 5% CO_2_ incubator under 37°C. SV40 MES-13 cells with good growth condition were divided into fivegroups. Cells in experimental group were incubated with HG (30 mM) [3] and different concentrations of DGT-containing serum (0, 5, 10 and 20%) for 72 h.

pcDNA-GAS5 was constructed by inserting GAS5 cDNA into pcDNA3.1 (catalog number: V79020, Invitrogen, U.S.A.). To detect the role of GAS5 on IκB kinase (IKK) protein level and the role of NF-κB on the proliferation of HG-induced mesangial cells and accumulation of extracellular matrix, pcDNA-GAS5, small interference RNA targeting GAS5 (si-GAS5), si-IKK or negative controls were synthesized by Sangon Biotech (Shanghai, China). SV40 MES-13 cells were seeded at 3 × 10^5^ cells/well in a six-well plate and cultured until 50% confluence, then transfected with 60 nmol si-GAS5, or 60 nmol pcDNA-GAS5, or 60 nmol si-IKK or negative controls into SV40 MES-13 cells using RNAfectin TRANSfection Reagent (catalog number: DP104, Tiangen, Beijing, China) according to the manufacturer’s instructions.

### Water-soluble tetrazolium salt WST-1 assay

SV40 MES-13 cell viability was detected by WST-1 assay [22]. SV40 MES-13 cells (control, treated with HG, pcDNA-GAS5, si-IKK) were seeded into 96-well plate with DMEM containing 10% FBS. Then, WST-1 (catalog number: C0035, Beyotime Biotechnology, Nantong, China) was added to the culture wells and the mixtures were incubated at 37°C for 2 h. The plates were read by a scanning multi-well spectrophotometer under a wavelength of 450 nm and a reference wavelength of 630 nm.

### Western blot analysis

Radio immunoprecipitation assay (RIPA) buffer was used to extract proteins from different treated SV40 MES-13 cells [19]. Then, the concentrations of the proteins were detected by BCA Protein Assay Ksmall interference RNA targeting GAS5 it (catalog number: 23250, Pierce Biotechnology). Protein samples were separated on 10% SDS/polyacrylamide gel electrophoresis (SDS/PAGE) gel and transferred to polyvinylidene-fluoride (catalog number: T2234, Invitrogen). Then, the membrane was blocked with 5% non-fat dried milk for 2 h. Anti-IKK (1:1000; catalog number: ab178870, Abcam, U.S.A.) and anti-β-actin (1:1000; catalog number: MA5-15739, Invitrogen, U.S.A.) were used as the first primary antibody, and the corresponding horseradish peroxidase-conjugated secondary antibody was added and incubated at room temperature for 1 h. Protein-antibody complexes were visualized by enhanced chemiluminescence system (ECL; catalog number: 35055, Thermo Scientific, U.S.A.).

### Quantitative real-time PCR

TRIzol reagent (Invitrogen) was used for isolating total RNA from SV40 MES-13 cells at different groups according to manufacturer’s instructions [23]. Then, SuperScript™ IV One-Step RT-PCR System (catalog number: 12594025, Invitrogen) was used for the preparation of cDNA. QuantStudio®3 Quantitative real-time PCR instrument (Applied Biosystems) was used to perform qRT-PCR reactions. A total fluid volume of 25 μl and TaqMan™ Fast Advanced Master Mix (catalog number: 4444556, Applied Biosystems) were used for the amplification. A two-step cycle protocol was used for detecting GAS5 expression. ABI Prism 7000 SDS software (Applied Biosystems) was used for analyzing the data. The relative expression of GAS5 was calculated by comparative method 2^−ΔΔ*C*^_t_.

### RNA pull-down

Biotin-labeled GAS5 was transcribed with the Biotin RNA Labeling Kits (catalog number: 11685597910, Roche, Switzerland) and T7 polymerase, and purified with TriPure Isolation Reagent (catalog number: 11667157001, Roche) [24].One miligram protein from SV40 MES-13 cells extracts was mixed with 50 pmol biotin-labeled RNAs, incubated with streptavidin agarose (catalog number: 20347, Thermo Scientific) and then subjected to Western blotting.

### Enzyme-linked immunosorbent assay

The protein expressions of LN, FN and Col IV in the culture medium were quantified by Mouse LN Enzyme-linked immunosorbent assay (ELISA) Kit (catalog number: D720371-0048, Sangon Biotech, Shanghai, China), Mouse FN ELISA Kit (catalog number: D720121-0048, Sangon Biotech, Shanghai, China) and Mouse COL4 ELISA Kit (catalog number: E-EL-M0317km, elabscience, Shanghai, China) according to the manufacturer’s instructions [19]. The absorbance was measured at 450 nm with a microplate reader.

### Luciferase assays

IKK wild-type and IKK mutant reporter vectors were constructed and inserted into the psiCHECK-2 (Promega, U.S.A.) [25]. The reporter plasmids (IKK wild-type or IKK mutant) together with si-GAS5 or si-control were transfected into SV40 MES-13 cells using Lipofectamine 2000 (catalog number: 11668019, Invitrogen) for 48 h for Luciferase reporter assay. Cells were collected to measure luciferase activity by dual Glo™ Luciferase Assay System (catalog number: E1910, Promega, U.S.A.).

### Statistical analysis

Statistic Package for Social Science 18.0 software (SPSS 18.0) was used for the data analysis, and the data were presented as mean ± standard deviation (SD). Comparisons between two groups or among multiple groups were analyzed by Student’s *t* test or one-way analysis of variance (ANOVA), with *P<*0.01 considered statistically significant.

## Results

### Effect of HG and DGTXT on the expressions of GAS5 and IKK

**Figure 1:**
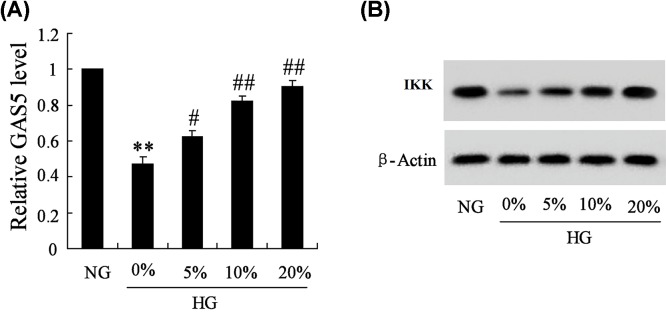
Mouse renal mesangial cells (SV40 MES-13) were treated with 5.6 mM glucose (NG), 30 mM glucose (HG)+DGTXT (0, 5, 10 and 20%) (**A**) The expressions of GAS5 in SV40 MES-13 cells were detected in NG, 0% HG, 5% HG, 10% HG, 20% HG groups. (**B**) Protein levels of IKKin NG, 0% HG, 5% HG, 10% HG, 20% HG groups. ** *P*<0.01, compared with NG group.^##^ *P*<0.01, compared with HG group.

To investigate the effect of HG and DGTXT on the expressions of GAS5 and IKK, we divided SV40 MES-13 cells into five groups. As shown in Figure 1A, HG down-regulated the expression of GAS5, while DGTXT up-regulated the expression of GAS5 dose-dependently, which indicated that DGTXT could increase GAS5 level. Meanwhile, HG down-regulated the expression of IKK, while DGTXT up-regulated the expression of IKK dose-dependently (Figure 1B), indicating that DGTXT could increase the expression of IKK, which meant DGTXT inhibited NF-κB signaling pathway.

### DGT participated in HG-regulated IKK expression and NF-κB activity via GAS5

**Figure 2:**
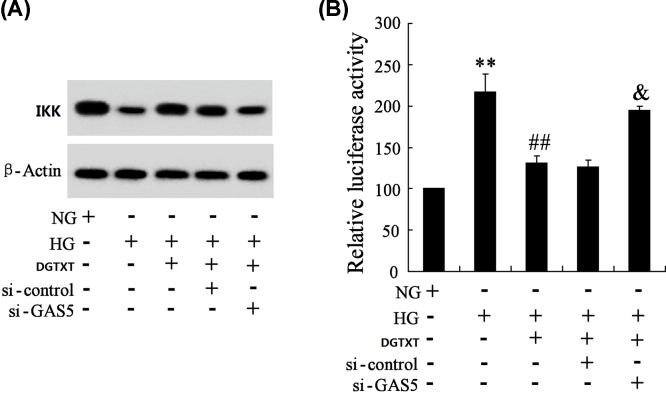
DGT participated in the expression of IKK and the activity of NF-κB regulated by HG via GAS5 (**A**) SV40 MES-13 cells were transfected with si-GAS5 or si-control. Then, SV40 MES-13 cells were divided into normal glucose (NG) group, HG group, HG+DGTXT (10%) group, HG+DGTXT (10%)+si-control group, HG+DGTXT (10%)+si-GAS5 group. The expressions of IKK were detected in NG group, HG group, HG+DGTXT (10%) group, HG+DGTXT (10%)+si-control group, HG+DGTXT (10%)+si-GAS5 group. (**B**) The luciferase activity of IKK was measured in NG group, HG group, HG+DGTXT (10%) group, HG+DGTXT (10%)+si-control group, HG+DGTXT (10%)+si-GAS5 group. ** *P*<0.01, compared with NG group. ^##^*P*<0.01, compared with HG group. ^&^*P*<0.01, compared with HG+ DGTXT+si-control group.

To find out how DGT participated in the expression of IKK and the activity of NF-κB regulated by HG, silencing GAS5 was used to observe the changes of IKK and NF-κB. In SV40 MES-13 cells treated with HG and 10% DGTXT, HG down-regulated the expression of IKK, and DGTXT reversed the down-regulation of IKK, while si-GAS5 cancelled the changes of IKK expression (Figure 2A). The activity of NF-κB was measured by luciferase assay. HG up-regulated the luciferase activity of NF-κB, DGTXT down-regulated the luciferase activity of NF-κB, while si-GAS5 cancelled the changes of NF-κB luciferase activity (Figure 2B). These results suggested that DGT participated in HG-regulated IKK expression and NF-κB activity via GAS5.

### Interaction between GAS5 and IKK in SV40 MES-13 cells

**Figure 3:**
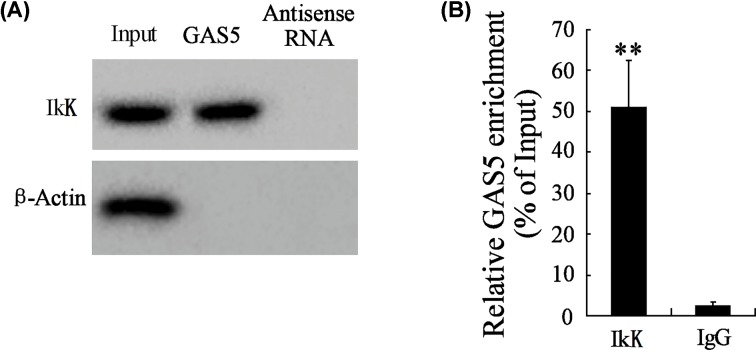
The interaction between GAS5 and IKK in SV40 MES-13 cells (**A**) RNA-pull down assay using *in vitro*-synthesized biotinylated GAS5. Precipitation reactions were conducted using streptavidin beads and then subjected to Western blotting. (**B**) Protein lysates were prepared and immunoprecipitated with IKK antibody or IgG. The RNA levels of GAS5 in immunoprecipitates were measured by real-time PCR. ** *P*<0.01, compared with IgG.

The interaction between GAS5 and IKK in SV40 MES-13 cells was detected by RNA pull-down assay. Precipitation reactions were conducted using streptavidin beads. Protein lysates were prepared and immunoprecipitated with IKK antibody or IgG. Real-time PCR was used to measure RNA levels of GAS5 in immunoprecipitates. According to Figure 3A,B, we found that GAS5 could bind to IKK, and they were positively related.

### GAS5 regulated the expression of IKK and the activity of NF-κB

**Figure 4:**
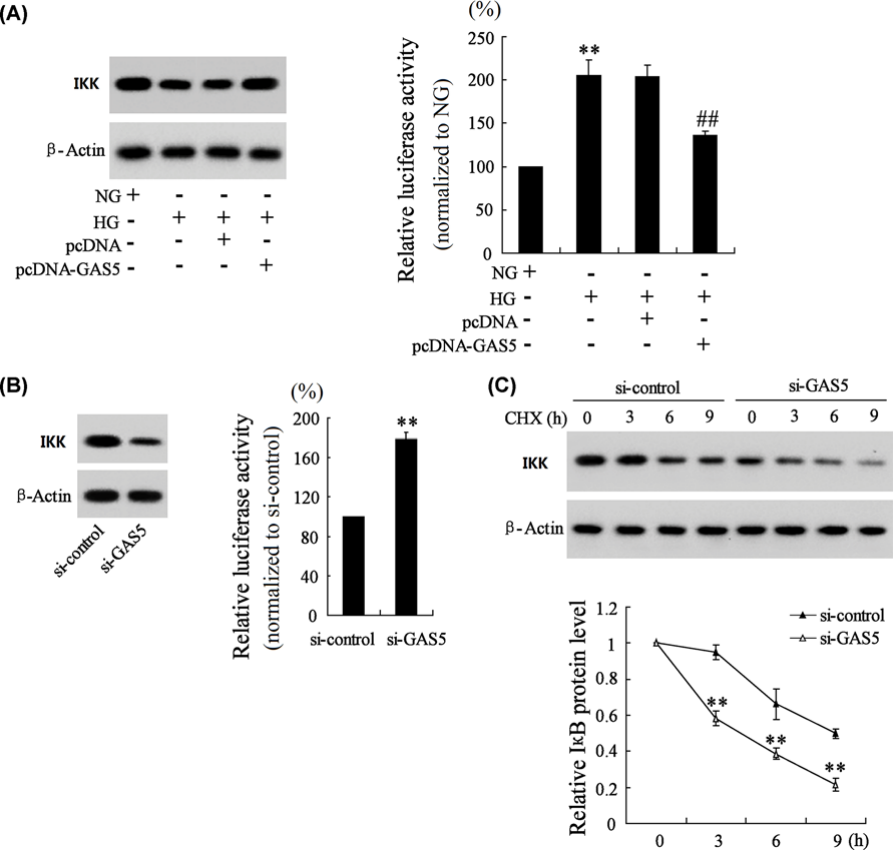
GAS5 regulated the expression of IKK and the activity of NF-κB (**A**) SV40 MES-13 cells were transfected with pcDNA or pcDNA-GAS5. Then, SV40 MES-13 cells were divided into NG group, HG group, HG+pcDNA group, HG+pcDNA-GAS5 group. The expression of IKK and the luciferase activity of NF-κB were detected in NG group, HG group, HG+pcDNA group, HG+pcDNA-GAS5 group. (**B**) SV40 MES-13 cells were transfected with si-control or si-GAS5. The expression of IKK and the luciferase activity of NF-κB were detected in si-control and si-GAS5 groups. (**C**) SV40 MES-13 cells were transfected with si-control or si-GAS5, then treated with Cycloheximide (CHX) for 0, 3, 6 and 9 h. The protein level of IKK was detected in each group. ** *P*<0.01, compared with NG or si-control. ^##^*P*<0.01, compared with HG+pcDNA.

Since GAS5 could bind to IKK, we speculated that GAS5 regulated the expression of IKK and the luciferase activity of NF-κB. To prove this assumption, we overexpressed and inhibited the expression of GAS5 to see the changes of IKK expression and NF-κB luciferase activity. As shown in Figure 4A, overexpression of GAS5 reversed HG-induced down-regulation of IKK expression and up-regulation of NF-κB luciferase activity. While si-GAS5 decreased the expression of IKK and increased the luciferase activity of NF-κB (Figure 4B). At the same time, si-GAS5 promoted the degradation of IKK (Figure 4C). These findings suggested that GAS5 was positively related with IKK, and negatively related with NF-κB signaling pathway.

### DGT inhibited the proliferation of HG-induced mesangial cells and accumulation of extracellular matrix via GAS5

**Figure 5:**
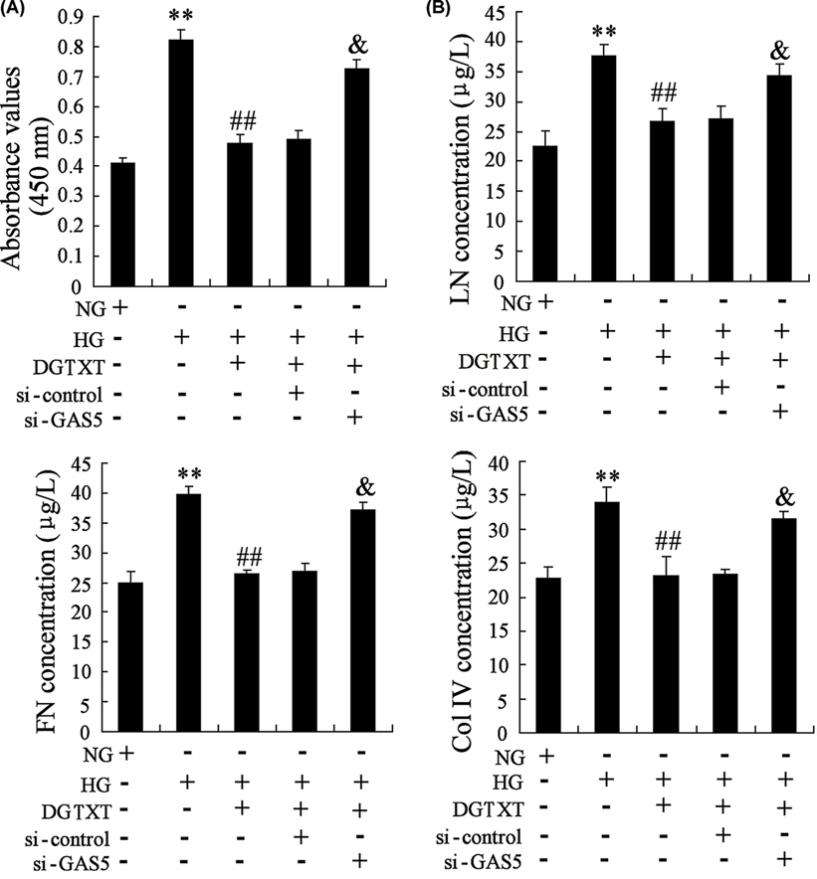
DGT inhibited the proliferation of HG-induced mesangial cells and accumulation of extracellular matrix via GAS5 SV40 MES-13 cells were transfected with si-control or si-GAS5. Then, SV40 MES-13 cells were divided into NG group, HG group, HG+DGTXT group, HG+DGTXT+si-control group, HG+DGTXT+si-GAS5 group. (**A**) The proliferation of SV40 MES-13 cells was detected in NG group, HG group, HG+DGTXT group, HG+DGTXT+si-control group, HG+DGTXT+si-GAS5 group. (**B**) The levels of LN, FN and Col IV were detected in NG group, HG group, HG+DGTXT group, HG+DGTXT+si-control group, HG+DGTXT+si-GAS5 group. ** *P*<0.01, compared with NG. ^##^*P*<0.01, compared with HG. ^&^*P*<0.01, compared with HG+DGTXT+si-control.

As shown in Figure 5A, HG promoted the proliferation of SV40 MES-13 cells, DGTXT inhibited the proliferation of SV40 MES-13 cells and GAS5 interference could reverse the inhibition effect. However, HG promoted extracellular matrix-related protein levels, such as LN, FN and Col IV and DGTXT inhibited the levels of LN, FN and Col IV (Figure 5B).

### GAS5 regulated the proliferation of HG-induced mesangial cells and accumulation of extracellular matrix through NF-κB pathway

**Figure 6:**
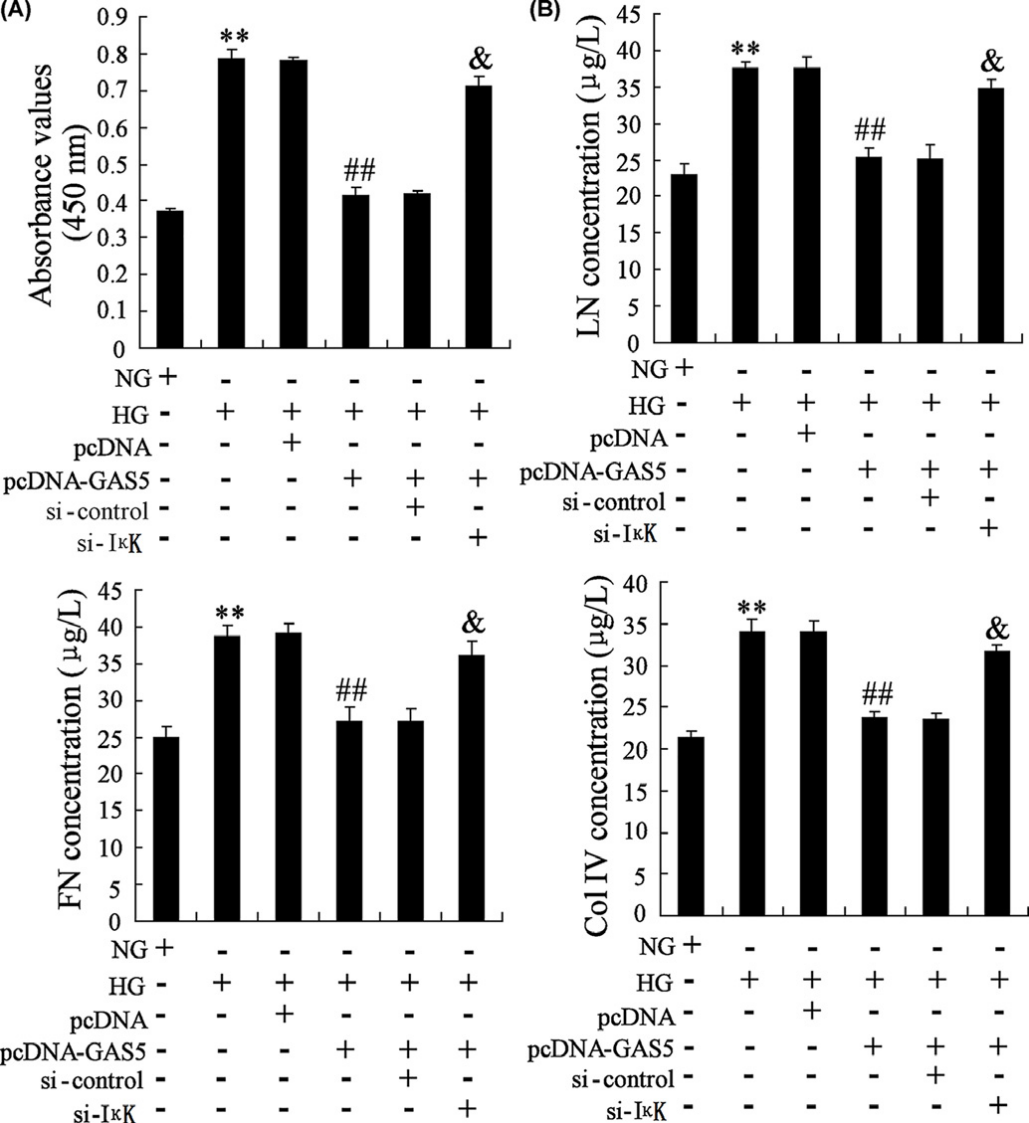
GAS5 regulated the proliferation of HG-induced mesangial cells and accumulation of extracellular matrix through NF-κB pathway SV40 MES-13 cells were transfected with pcDNA, or pcDNA-GAS5, or pcDNA-GAS5+si-control, or pcDNA-GAS5+si-IKK. Then, SV40 MES-13 cells were divided into NG group, HG group, HG+pcDNA group, HG+pcDNA-GAS5 group, HG+pcDNA-GAS5+si-control group, HG+pcDNA-GAS5+si-IKK group. (**A**) The proliferation of SV40 MES-13 cells was detected in NG group, HG group, HG+pcDNA group, HG+pcDNA-GAS5 group, HG+pcDNA-GAS5+si-control group, HG+pcDNA-GAS5+si-IKK group. (**B**) The levels of LN, FN and Col IV were detected in NG group, HG group, HG+pcDNA group, HG+pcDNA-GAS5 group, HG+pcDNA-GAS5+si-control group, HG+pcDNA-GAS5+si-IKK group.** *P*<0.01, compared with NG. ^##^*P*<0.01, compared with HG+pcDNA. ^&^*P*<0.01, compared with HG+ pcDNA-GAS5+si-control.

To figure out whether GAS5 regulated the proliferation of HG-induced mesangial cells and accumulation of extracellular matrix through NF-κB pathway, we overexpressed GAS5 and interferred IKK to see the changes of cell viability. HG promoted the proliferation of SV40 MES-13 cells, overexpression of GAS5 inhibited HG-induced proliferation and silencing IKK cancelled the effect of GAS5 overexpression (Figure 6A). In addition, HG promoted extracellular matrix-related protein LN, FN and Col IV levels. Overexpression of GAS5 inhibited HG-induced up-regulation of LN, FN and Col IV levels. And IKK interference cancelled the effect of GAS5 overexpression (Figure 6B).

## Discussion

DN is one of the most important long-term complications of diabetes and the main cause of end-stage renal disease, which often has a progress of 10–20 years [26]. It is reported that it occurs in approximately 20–40% of patients with type two diabetes mellitus [27], and the incidence and prevalence of DN is increasing worldwide. The pathological characteristics of DN included the proliferation of mesangial cells and accumulation of extracellular matrix [28]. Ke et al [29] demonstrated that in glomerular mesangial cells, HG induced an increased synthesis of extracellular matrix proteins, including LN, FN and Col IV. Moreover, these pathological changes would promote progressive diabetic glomerulosclerosis and renal function damage [30]. Therefore, it is necessary to find an effective strategy to inhibit the progress of DN.

DGT is an ancient Chinese herbal decoction that has been demonstrated to possess hematopoietic function and has important values in glucose homeostasis, which may be a valid therapy for the control of diabetes and its complications [31]. Besides, protective effects of DGT on renal glomerular mesangium and renal function in STZ-induced rats were also explored [28,32]. Previous report has concluded that DGT could attenuate extracellular matrix components, such as FN or Col IV [9]. In glomerular mesangial cells, researchers have proved that DGT inhibited cell proliferation and the expression of LN, FN and Col IV. On the one hand, the levels of LN, FN and Col IV can be changed by the increase of cell numbers. On the other hand, they can be changed by the secretion ability of single cells. DGT inhibited the proliferation of HG-induced extracellular matrix accumulation by regulating GAS5/NF-κB might be related with the two aspects. In the present study, we proved that DGT could inhibit the viability of HG-induced SV40 MES-13 cells and decrease the concentrations of LN, FN and Col IV, which was consistent with our assumption. However, the studies of DGT on the treatment of DN are still lacked, and the exact mechanism is still unclear.

According to previous reports [11–13], we speculated that DGT might inhibit the proliferation of mesangial cells and accumulation of extracellular matrix via NF-κB pathway. In addition, lncRNA GAS5 was negatively related with NF-κB and could express in renal cells [16,18], therefore, we assumed that DGT might inhibit the proliferation of mesangial cells and accumulation of extracellular matrix via GAS5/NF-κB. In the present study, we first observed that DGT up-regulated the expression of GAS5 and IKK dose-dependently. Then, we found DGT participated in the expression IKK and the activity of NF-κB via GAS5, and proved that lncRNA GAS5 was positively related with IKK in SV40 MES-13 cells, thus proving lncRNA GAS5 was negatively related with NF-κB.

In conclusion, the findings of the present study clearly show remarkable renoprotective effects of DGT. The mechanism underlying this protective effect is regulating proliferation of mesangial cells and accumulation of extracellular matrix via GAS5/NF-κB. However, more studies are needed in the future to clarify more specific mechanism of DGT on the treatment of DN.

## Abbreviations

Col IV: collagen IV
DGT: Danggui buxue tang
DN: diabetic nephropathy
ELISA: enzyme-linked immunosorbent assay
FBS: fetal bovine serum
FN: fibronectin
GAS5: growth arrest specific transcript 5
IKK: IκB kinase
LN: laminin
NF-κB: nuclear transcription factor-κB
si-GAS5: small interference RNA targeting GAS5
STZ: streptozotocin
